# Endovascular Treatment of a Ruptured Splenic Artery Aneurysm using Amplatzer^®^ Vascular Plug

**Published:** 2009-03

**Authors:** UD Manian, H Badri, PE Coyne, CA Nice, HY Ashour, V Bhattacharya

**Affiliations:** 1 *Department of Vascular Surgery, Queen Elizabeth Hospital, Gateshead, UK;*; 2 *Department of Interventional Radiology, Queen Elizabeth Hospital, Gateshead, UK*

**Keywords:** splenic artery aneurysm, rupture, endovascular embolisation

## Abstract

Splenic Artery Aneurysms are commonly detected incidentally and can present acutely as a source of intra-abdominal catastrophe. Management options include both surgical and endovascular repair. The role of endovascular repair in an haemodynamically stable acute rupture is undefined and the use of Amplatzer^®^ Vascular Plug has not to our knowledge been reported.

## INTRODUCTION

Splenic artery aneurysms are the third commonest intra-abdominal aneurysms seen after aortic and iliac aneurysms (1). Splenic artery aneurysms have a natural tendency to grow until they rupture. Current indications for repair are based on the risk of aneurysm rupture. Indications include a diameter >20 mm, pregnancy, symptomatic aneurysms and patients undergoing liver transplant.

Splenic artery aneurysms have an incidence of 0.1% in the general population. This rises to 10% in those over 60 and up to 50% in those with portal hypertension (3). Autopsy incidence has also been shown to be between 0.2-2% in all age groups and 10.4% above the age of 60(8). Risk factors for splenic artery aneurysms include portal hypertension, systemic hypertension, multiparity and arteriosclerosis (4). The majority of splenic artery aneurysms are asymptomatic being incidentally detected on ultrasound or CT, but some patients can present with left upper quadrant pain. Importantly, 10% of cases present with a ruptured aneurysm, with signs of an acute abdomen or circulatory shock. These patients have a significant risk of death estimated between 10-25% in those who are not pregnant, worsening to 70% during pregnancy (2, 4).

Endovascular management of splenic artery aneurysms is increasingly being used over surgery in the elective setting. However, in the emergency setting, surgery is still the most commonly used treatment modality.

We present a case of successful endovascular repair of a ruptured splenic artery aneurysm using Amplatzer^®^ Vascular Plug.

## CASE

A 63-year-old male with known hypertension, presented to his family practitioner with a two-week history of left sided abdominal pain radiating into his back. An outpatient ultrasound scan (USS) was arranged. In the ultrasound department he developed stabbing left flank pain. He was haemodynamically stable but examination confirmed a pulsatile mass in the left upper quadrant.

**Figure 1:**
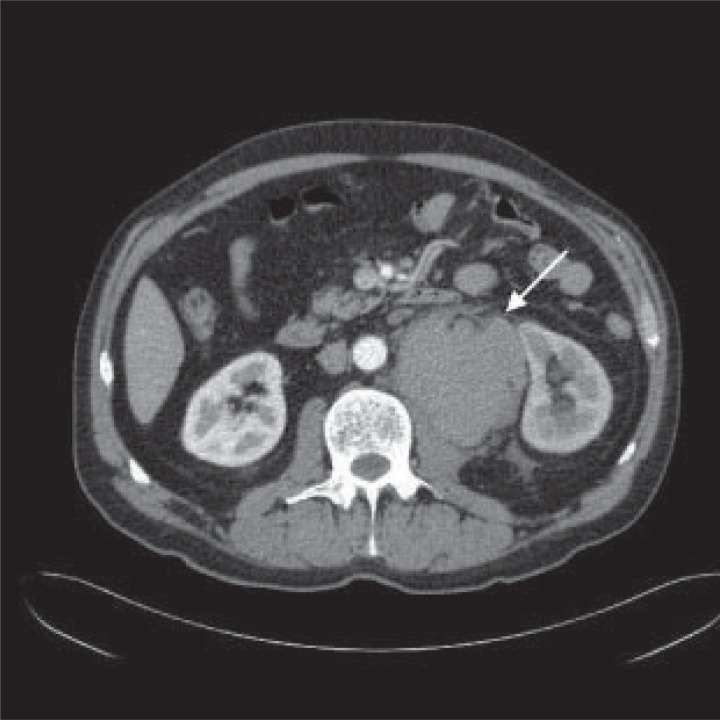
CT scan shows retroperitoneal haematoma (arrow) between left psoas muscle and left kidney.

USS revealed a 100 × 85 × 57 mm haematoma in the retroperitoneum posterior to the left kidney. An emergency CT scan was done which identified a 62 mm mid splenic artery aneurysm. This extended inferiorly to the haematoma confirming rupture of the aneurysm (Fig. 1).

Urgent endovascular treatment was undertaken. Preliminary angiography showed a moderately tortuous splenic artery, with a large saccular aneurysm that had a wide neck unsuitable for stent graft repair. Occlusion of arterial outflow and inflow was therefore performed to isolate the aneurysm sac.

## AMPLATZER^®^ VASCULAR PLUG TECHNIQUE

**Figure 2:**
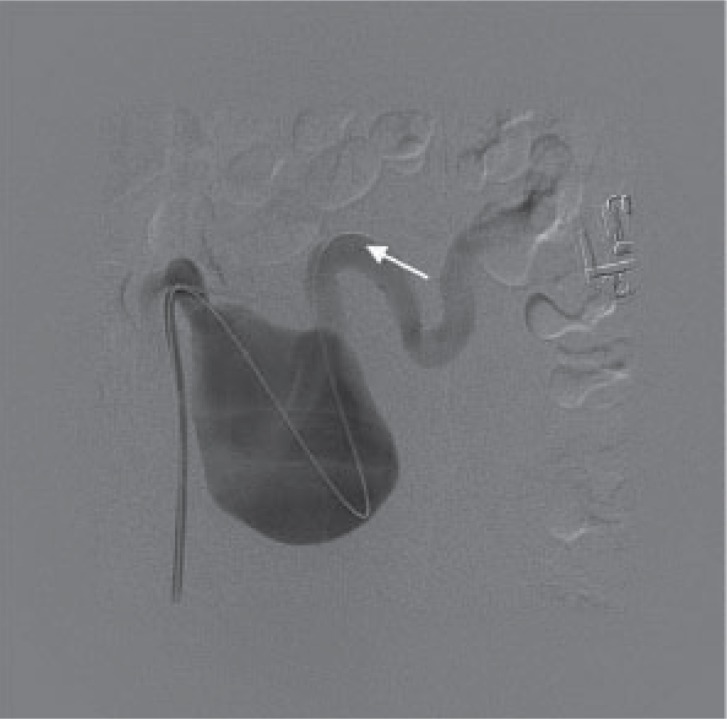
Splenic artery (arrow) distal to aneurysm catheterized using hydrophilic guidewire.

Under local anaesthetic the right common femoral artery was punctured and a 6FG vascular sheath inserted. The distal splenic artery was selectively catheterised with a hydrophilic wire. This was subsequently exchanged for a 1cm floppy tip super stiff wire (Fig. 2).

**Figure 3:**
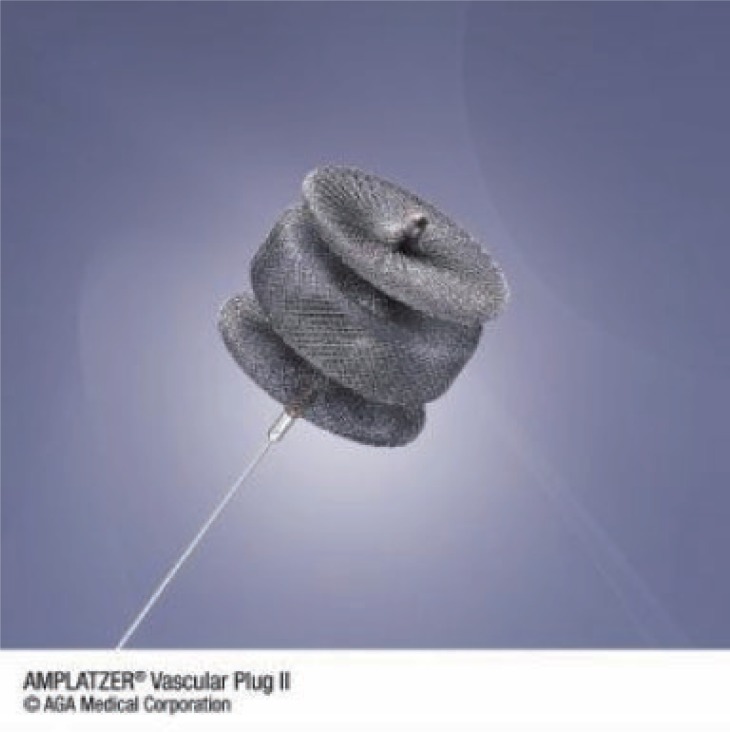
Amplatzer ® vascular plug pre-expansion.

**Figure 4:**
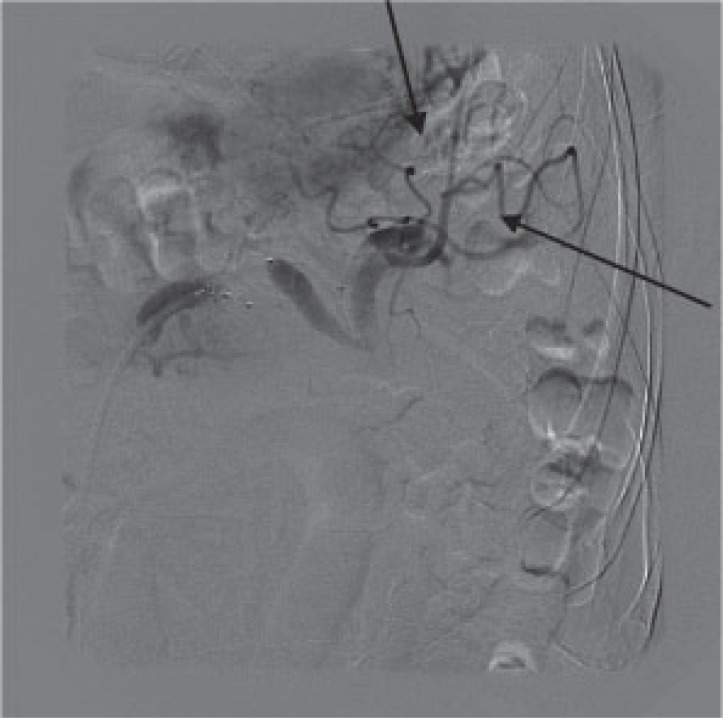
Successful exclusion of aneurysm with distal perfusion via collaterals (arrows).

A 7FG destination (Terumo Medical Corporation, New Jersey, USA) vascular sheath was advanced into the distal splenic artery and the distal artery embolised with a 12 mm diameter Amplatzer^®^ vascular plug (AGA Medical, Minnesota, USA) (Fig. 3). The sheath was then withdrawn proximal to the aneurysm and the proximal vessel occluded with a 10 mm diameter Amplatzer^®^ vascular plug. Good perfusion of the spleen and pancreatic tail was shown via collateral arteries (Fig. 4).

**Figure 5:**
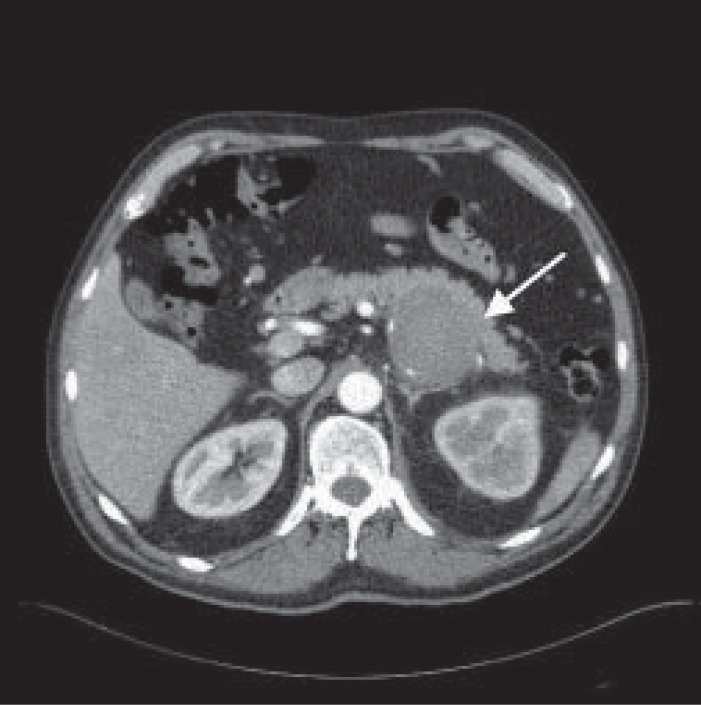
Follow up CT angiogram shows no flow in the aneurysm sac (arrow).

The patient was monitored on the High Dependency Unit post-procedure. CT Angiography was performed two days later and this showed that the splenic artery aneurysms remained successfully excluded from the circulation (Fig. 5). The associated retroperitoneal haematoma was static in appearance. The majority of the splenic parenchyma was well perfused, with around 10-20% estimated loss. The patient was successfully discharged home 3 days later.

**Figure 6:**
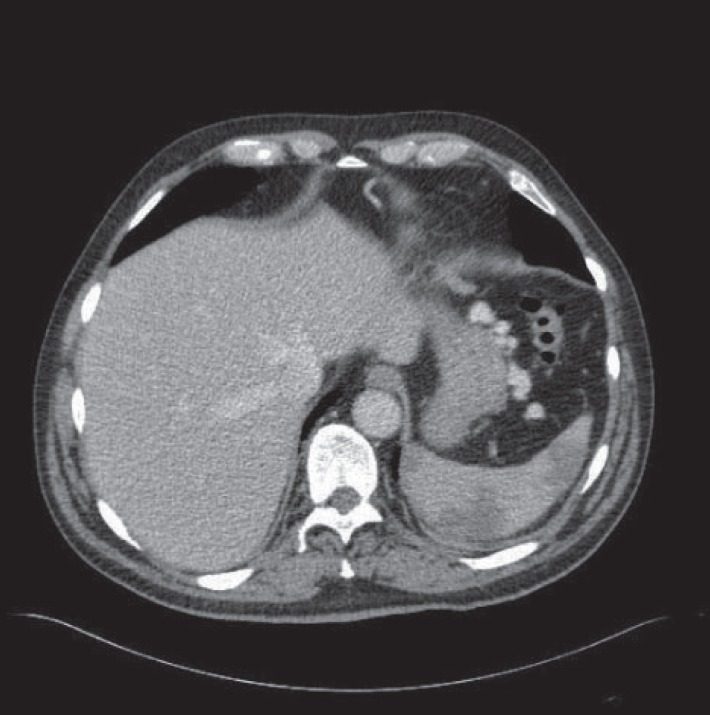
Computed Tomography (portal venous phase images) showing small segmental splenic infarcts (10-20% volume).

At follow up three weeks later, he remains well with no complications. Followup CT scans show no flow in the aneurysm sac and small segmental splenic infarcts (Fig. 6).

## DISCUSSION

Traditionally treatment of splenic artery aneurysms was done surgically with ligature of the splenic artery and splenectomy. Rare cases of splenic artery aneurysm resection with vessel reconstruction have been reported (5). Surgical repair is associated with a high degree of morbidity resulting from splenectomy and distal pancreatectomy as well as significant mortality (5% in elective cases and 40% in emergency cases (4)). As a consequence endovascular repair of splenic artery aneurysms are being increasingly employed.

Endovascular aneurysm repair encompasses insertion of a stent graft or coil embolisation. In patients with suitable anatomy stent-graft repair allows visceral aneurysm exclusion with preservation of arterial flow. Coil embolization provides an excellent alternative for tortuous aneurysm occlusion. Conventional coils can be placed to block a narrow-necked splenic artery aneurysm allowing continued flow through the artery. Wide-necked splenic artery aneurysms pose more problems however. Current practice is to deploy several coils both distal and proximal to the splenic artery aneurysm to obtain complete devascularisation, with blood flow to the spleen maintained by collateral vessels. Precise placement of the coils can be difficult, with multiple coils being needed to occlude a single aneurysm. The use of the Amplatzer^®^ Vascular Plug (AVP) for exclusion of splenic artery aneurysm repair has not previously been documented. Initially indicated for the correction of cardiac septal defects, reports of successful use in peripheral artery aneurysm repair have emerged (6). The AVP is a flexible, self-expanding cylindrical device and can be used with a wide variety of aneurysm morphologies. The location of the AVP can be tested before final placement ensuring precise positioning and complete occlusion of the aneurysm. The AVP is appropriately sized for medium blood vessels such as the splenic artery. Current reports demonstrate fewer complications with AVP over standard metal coils (6, 7).

## SUMMARY

Endovascular repair of splenic artery aneurysm is less invasive, can be done under local anaesthetic has shorter hospital stays and most importantly preservation of splenic function compared to surgery. It can also be used selectively in an emergency setting in patients who are haemodynamically stable with rupture of a splenic artery aneurysm.

## CONFLICTS OF INTEREST

The authors declare that no conflicting interests exist.
